# Harnessing Cattail Biomass for Sustainable Fibers and Engineered Bioproducts: A Review

**DOI:** 10.1002/gch2.202400183

**Published:** 2024-11-24

**Authors:** Mahmuda Parvin, Md Shadhin, Marzia Dulal, Mashiur Rahman

**Affiliations:** ^1^ Department of Biosystems Engineering University of Manitoba Manitoba R3T 2N2 Canada; ^2^ Department of Textile Engineering Management Bangladesh University of Textiles Tejgaon Industrial Area Dhaka 1208 Bangladesh

**Keywords:** carbon dioxide sequestration, degumming, extraction methods, lignocellulosic fibers, typha

## Abstract

Cattail (*Typha*), a wetland plant, is emerging as a sustainable materials resource. While most of the *Typha* species are proven to be a fiber‐yielding crop, *Typha latifolia* exhibits the broadest leaf size (5–30 mm), yields highest amount of fiber (≈190.9 g), and captures maximum CO_2_ (≈1270 g). Alkaline retting is the most efficient degumming process for cattail fibers to achieve maximum fiber yield (30%–46%). Cattail leaves exhibit a distinctive bionic structural model consisting of epidermis and leaf blade at macro level and non‐diaphragm aerenchyma, fiber cables, partitions, and diaphragms at micro level. Cattail fibers hold promise to be utilized as a high‐performance composite part and as efficient energy storage devices in clean energy vehicles. The former is attributed to their lower density (≈1.26–1.39 gm/cm^3^) and higher tensile modulus (≈66.1 GPa after treatment), while the latter is attributed to their porous structure and chemical stability. Therefore, integrating the knowledge of plant biology and materials chemistry is crucial for enhancing fiber characteristics and producing engineered bioproducts. The environmental benefits of cattails, degumming methods, leaf and fiber structures, their properties and applications is reviewed. Finally, it discussed future research directions aimed at developing bioengineered, biodegradable products from it with minimal environmental impact.

## Graphical abstract

Integrating plant biology and materials chemistry is vital for enhancing cattail fiber characteristics and developing engineered bioproducts. This review explores cattails' environmental benefits, including carbon sequestration, alongside degumming methods for fiber extraction. It examines leaf and fiber structures, their properties, and diverse applications, highlighting cattails' potential as a sustainable resource for sensors, energy storage, textiles and biocomposites.

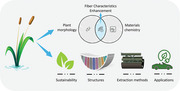

## 1 Introduction

Natural plant fibers, such as cotton, jute, flax, hemp, sisal, and kenaf, are being studied as renewable resources and eco‐friendly alternatives to energy‐intensive synthetic materials that emit greenhouse gases (GHG), produce microplastics, pollute the environment, and contaminate freshwater systems during their production as well as disposal.^[^ ^1^, ^2^, ^3^, ^4^, ^5^ ^]^ In addition to sustainability, they offer cost‐effectiveness, lightweight, recyclability, and widespread distribution, which is driving the interest from non‐biodegradable fossil‐based materials to these biobased resources.^[^ ^6^, ^7^ ^]^ However, the use of these biobased materials is restricted due to the limited supply constraint.^[^ ^8^, ^9^, ^10^ ^]^ Moreover, plant‐based fibers require agricultural activities at particular climatic conditions, involve pesticide and insecticide application, and utilize water, energy, and other natural resources for crop production, which consequently depletes natural resources, reduces soil fertility, increases the overall carbon and water footprint, and impacts food supplies.^[^ ^11^, ^12^, ^13^ ^]^ These factors, together with the cost and expertise required to manage specialized harvesting machines and the longer lead time for vegetation,^[^ ^14^ ^]^ disrupt their feasibility. Considering the above factors, it is necessary to locate abundant and cost‐effective fiber plants and utilize waste stream resources to achieve sustainable development goals. Numerous wetland plants such as *Typha latifolia, Salix petiolaris, Typha angustifolia, Rubus hispidus, Rhamnus frangula, Fraxinus nigra, Onoclea sensibilis*, and *Lythrum salicaria* are potential sources of waste biomass. These plants are highly productive, naturally growing,^[^ ^15^ ^]^ widely available and rich in cellulose content in their chemical composition.^[^ ^16^ ^]^

Cattail is a wetland plant that can be harnessed as a natural resource for industrial applications. For instance, cattail species‐ *Typha latifolia* are gaining attention as a renewable and sustainable resource, particularly in eco‐friendly construction and material innovation. Recent studies highlight their potential as industrial fibers due to their high quality and abundance, indicating a promising avenue for sustainable materials in building applications.^[^ ^17^ ^]^ Research indicates that cattails can be processed into various forms, including nanocellulose, which demonstrates impressive thermal stability and mechanical properties, making them suitable for advanced composite materials.^[^ ^18^ ^]^ Nanocellulose extracted from cattail through hydrogen peroxide‐acid treatment demonstrated high thermal stability and good light transmission, with a spider‐web‐like structure and diameters between 5 and 20 nm.^[^ ^19^ ^]^ Notably a recent study found that cattail fibers maintained consistent mechanical properties throughout multiple harvests within a single growing season, attributed to key morphological and molecular changes. The presence of calcium oxalate plates (COPs), along with specific elemental compositions and infrared peaks (notably at 2360, 1635, 2920, and 3430 cm^−1^), demonstrated fiber strength and cellulose content. The findings show cattail fibers’ potential in sustainable composite material applications due to their stable mechanical properties across different growth stages.^[^ ^20^ ^]^ Furthermore, the resilience of cattails in diverse wetland environments underscores their ecological significance and adaptability, promoting their role in sustainable practices that align with environmental conservation goal.^[^ ^15^, ^21^ ^]^ It flourishes naturally in wetland environments without any agricultural practices, thereby reducing the cost of seed, cultivation, irrigation, and CO_2_ emissions.^[^ ^22^ ^]^ However, the proliferation of cattail plants requires meticulous oversight to sustain ecological balance.^[^ ^23^, ^24^, ^25^, ^26^ ^]^ Carbon dioxide (CO_2_) emissions are partially offset by the biofuel feedstocks provided by cattails.^[^ ^27^ ^]^The production of cattails helps reduce CO_2_ emissions from peatlands.^[^ ^28^ ^]^ *Typha* development can be beneficial in preventing subsidence and helps to reduce CO_2_ during growth. It was reported that emission from fertilizer (nitrogen, phosphorous, potassium, and sulphur) used during crop production of flax, hemp, and kenaf is 51.03, 129.19, and 101.53 kg CO_2_ /tonne stalk, respectively.^[^ ^29^ ^]^ In contrast, cattail requires no additional fertilizer or pest control (pesticides or herbicides).^[^ ^11^, ^30^ ^]^ Cattails were investigated to obtain fibers from leaves and seeds. Cattail biomass and fibers are widely being utilized as textiles and biocomposites,^[^ ^2^ ^]^ biofuels,^[^ ^31^ ^]^ energy storage,^[^ ^32^ ^]^ healthcare,^[^ ^33^ ^]^ sound absorption^[^ ^34^ ^]^ and so forth.

Compared to typical biomass fibers, cattail fibers yield higher production and lower density, and comparable mechanical properties, making them an excellent choice for engineered bioproducts. This is because the entire leaf turns into fibrous material. Cattail fibers show consistent mechanical properties across multiple harvests, attributed to molecular changes, such as the presence of COPs and cellulose content confirmed by specific infrared peaks. Additionally, the study investigated fiber delignification, which yielded 6.75 g l^−1^ of lignin while retaining up to 60% tensile strength.^[^ ^35^ ^]^ Cattail leaves are characterized by unique structural model and COPs. Knowledge of the former is required for product engineering. However, no previous study has provided a hierarchal structural model of cattail leaves yet. Removal of COPs in fibers causes a reduction in fiber density,^[^ ^22^ ^]^ which should result in an increase in specific properties and a decrease in the weight of the composite parts manufactured from cattail fibers. Therefore, cattail fiber surface engineering is essential for enhancing material performance and developing knowledge for design and production of bioproducts.

Moreover, a comparative study between Waste Biomass Fibers (WBFs) like cattail, canola, pineapple, and banana leaves and other Biomass Fibers (BFs) including flax, hemp, kenaf, jute, and bamboo in a sustainability evaluation shows WBFs routinely fared better than BFs; they scored a perfect 16 versus BFs' 9.^[^ ^29^ ^]^ Cattail is lighter (density ≈1.26 – 1.39 gm cm^3^) than traditional bast fibers, such as flax (density ≈1.54 gm cm^3^) and hemp (density ≈1.5 gm cm^3^).^[^ ^3^ ^]^ Hence, it could be a potential substitute for conventional biomass fibers. Additionally, using cattails in the industry can help with wetland weed problems and efficiently repurpose industry growing space. Cattail fibers provide an eco‐friendly and adaptable alternative for a range of applications due to their characteristics such as low carbon emissions, cost‐effectiveness, and recyclability.^[^ ^3^, ^36^, ^37^ ^]^

The research gap lies in the untapped potential of cattail fibers across various industrial applications, despite previous studies on fiber extraction and characterization. While past research has explored some methods for extracting fibers from *Typha*, there is a lack of comprehensive understanding of its species diversity, optimized extraction processes, and detailed structure‐property relationships. This study aims to provide a comprehensive review of *Typha*, covering its species, fiber extraction techniques from different plant parts, process optimization, and the structure and morphology of its leaves and fibers. Additionally, the environmental implications of using cattail fibers as a sustainable material have not been fully explored, especially when compared to conventional biomass sources. This study addresses these gaps by offering an in‐depth review of the entire lifecycle of cattail fibers, from species identification and fiber extraction techniques to material characterization and industrial applications. Furthermore, it focuses on optimizing processes to enhance fiber properties for specific applications while assessing the environmental benefits, such as reduced carbon emissions and resource efficiency. By advancing knowledge in these areas, the study aims to promote responsible consumption and sustainable material development, offering a practical pathway to reduce dependence on synthetic fibers and minimize environmental impact. With proper management, *Typha*, a resilient and abundant resource, offers the potential to expand the supply chain, transform agricultural waste into valuable products, and contribute to a more sustainable, eco‐friendly future. The distinctiveness of this study lies in its holistic approach to unlocking the untapped potential of *Typha* fibers, fostering progress toward a truly sustainable planet through CO_2_ sequestration, waste reduction and renewable technology.

## 2 The Growth of Cattails: The Adaptable *Typha* in Aquatic Ecosystems

**Figure 1:**
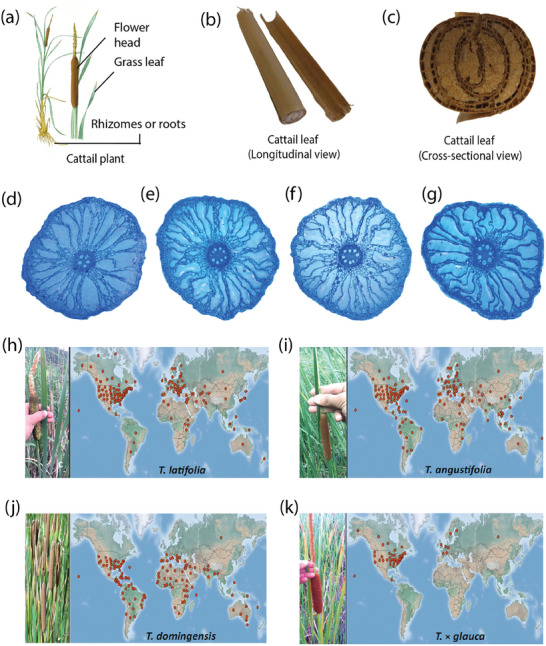
The growth of Cattails. a) Different parts of cattail plant b) longitudinal view of the cattail leaf; b) cross‐sectional view of the cattail leaf (b‐c: reproduced with permission.^[^ ^41^ ^]^ Copyright 2011 Elsevier B.V.). Transverse sections of the pilifer zone of Typha roots grown across various water availabilities d) waterlogged, e) field capacity, f) 75% field capacity, g) 50% field capacity (d‐g: reproduced with permission.^[^ ^48^ ^]^ © 2020 Elsevier B.V. Different Typha species across various locations (red dots) North America h) T. latifolia, i) T. angustifolia, j) T. domingensis, and k) T. × glauca (h‐k: adapted.^[^ ^27^ ^]^ unrestricted permission).

*Typha* is widely regarded as an invasive native plant in aquatic environments across the globe, particularly in North America.^[^ ^38^, ^39^ ^]^ This perennial wild herb part of the *Typhaceae* family, grows back year after year due to the stored energy in its rhizomes.^[^ ^5^, ^21^, ^36^, ^40^, ^41^ ^]^ Cattail includes species as *typhalatifolia*, calceolaria, wild candle, *typhaangustifolia*.^[^ ^37^ ^]^ Other traditional names of *Typha* have been mentioned: reed mace, cattail‐flag, flag tule, water torch, candlewick, punks, corn dog grass, elephant grass, bulrush, cumbungi, raupo, hogla, apu, sambu, rambaan, gond and pataar.^[^ ^40^, ^42^, ^43^ ^]^ The cattail plant (**Figure** **1**a) consists of three main parts: massive flat blade‐like grass leaves grown in spring; a cylindrical oval flower head; and rhizomes or roots.^[^ ^44^, ^45^ ^]^ Cattail has thick, ribbon‐like linear leaves and its underground stem (Figure 1a), similar to the leaves, and grows from thick, creeping rhizomes both having a spongy cross‐section due to the presence of air channels.^[^ ^41^ ^]^ The longitudinal and cross‐sectional views of the cattail leaves are shown in Figure 1b,c, respectively.

Cattail plants, forming flower heads from May and June and sometimes up to late July,^[^ ^39^ ^]^ are monoecious, with male and female flowers in one cluster.^[^ ^27^ ^]^ The male spike, yellow‐brown, releases tiny nutlets or pollen estimated between 280–420 Million,^[^ ^46^ ^]^ 200 000^[^ ^42^ ^]^ and 20 000–700 000,^[^ ^27^ ^]^ while the female spike is dark brown or reddish,^[^ ^42^ ^]^ forming a velvety, firm, cylindrical fruiting body. Upon ripening, the male spike releases pollen easily carried by wind, aiding seed dispersal., Seedlings develop from nutlets that germinate in water, leading to clonal plant growth from rhizomatous roots under various water conditions.^[^ ^39^, ^47^ ^]^ Figure 1d–g illustrate the transverse section of the *Typha* roots developed under various water conditions, such as waterlogged, field capacity, 75% field capacity, and 50% field capacity, respectively.

The word “cattail” originated in 1548 derived from the Greek word typhe (linguistically related to typhon, typhoon, and typhus), which means cloud, smoke, bulrush, monsters, storms, and diseases. Cattail (“a cat's tail”) describes how the plant looks when its seeds are released.^[^ ^45^, ^46^ ^]^ *Typha* has ≈40 species,^[^ ^27^ ^]^ includes prominent North American representatives: *Typha latifolia L*. (broadleaf cattail), *Typha angustifolia L*. (narrowleaf cattail), and *Typha domingensis Persoon* (southern cattail), along with thehybrid species *T. × glauca Godr*.,a cross of *T. latifolia* and *T. angustifolia*.^[^ ^27^, ^39^, ^46^, ^49^, ^50^ ^]^ Although the common name *cattail* is frequently used to refer to the entire genus *Typha* in most of the literature, *Typha latifolia L*. represents the word as common cattail.^[^ ^46^, ^51^ ^]^ The word ”lati“ means ”broad“ or ”wide,“ and the word “folia” refers to the plant's leaf width. Confusion arises between the common cattail (*T. latifolia L.)* and southern cattail (*T. angustifolia*) mainly in leaf width and color.^[^ ^46^ ^]^ Figure 1h–k demonstrates the worldwide distribution map of the well‐known species of *Typha*.^[^ ^27^ ^]^

Different species of Typha plant, such as *T. latifolia*, *T. angustifolia*, *T. domingensis*, are adapted to different geographical regions, as demonstrated in **Table** **1**. The botanical classification of these species together with the plant specifications, such as height, breadth, type and color of leaves, and number of leaves, are also summarized in Table 1. *T. domingensis* tends to grow taller (up to 4 m) than those of *T. latifolia* (≈1.2–3 m) and *T. angustifolia* (≈1.5–2.5 m). However, *T. latifolia* has the broadest leaf size (≈5–30 mm) of all three species. Cattail populations are found all over the world, in humid and dry climates, as well as tropical and temperate zones, demonstrating tolerance to changing climatic conditions dominating diverse aquatic locations.^[^ ^46^ ^]^ Wet or saturated soil is suitable for the growth of cattail such as swamplands, slow‐moving shallow waters, marshes, wet soil, ponds, dam's borders, low banks of freshwater ponds, canals, rivers, drainage ditches, and other freshwater or slightly brackish habitats.^[^ ^39^, ^41^, ^42^, ^49^ ^]^ Despite being a freshwater aquatic plant, cattails can withstand some salinity and acidity. While *Typha angustifolia* usually grows in more unstable and salty conditions, *Typha latifolia* can be found in comparatively undisturbed habitats.^[^ ^39^ ^]^ In addition, *Typha* can withstand regular flooding, deficient soil, and elevated levels of lead, zinc, copper, and nickel. Due to anthropogenic‐related changes to wetland hydrology and nutrient loads, distribution, and abundance of *Typha* in wetland ecosystems has expanded in recent decades, especially in North America (Figure 1h–k).^[^ ^27^ ^]^

**Table 1:** Overview of Typha species: geographical region, botanical classification, and plant specifications.

Species name	Distribution	Classification	Plant height [m]	Type and color of leaves	Number of leaves	Width of leaves [mm]
*T. latifolia* L.	North America, Newfoundland, Alaska, British Columbia, Mexico, Guatemala, The British Isles, Europe, Asia, Syria, Palestine and Japan.^[^ ^42^ ^]^	Kingdom: Plantae Subkingdom: Tracheobionta Super division: Spermatophyta Division: Magnoliophyta Class: Liliopsida Subclass: Commelinidae Order: Typhales Family: Typhaceae Genus: Typha L.^[^ ^43^ ^]^	1.2–2.5 m^[^ ^42^ ^]^	Broad‐leaved, light green^[^ ^42^ ^]^	12–16^[^ ^42^ ^]^	5—30 mm^[^ ^42^ ^]^
			3 m^[^ ^46^ ^]^		4‐12^[^ ^36^ ^]^	8 to 20 mm^[^ ^46^ ^]^
			4–9 feet^[^ ^43^ ^]^	Pale or grayish‐green leaves are flat 80 to 120 cm long^[^ ^46^ ^]^		10–27 mm^[^ ^36^ ^]^
			2.5–5 m^[^ ^49^ ^]^	Light green color^[^ ^36^ ^]^		
			3.1 m^[^ ^36^ ^]^			
*T. angustifolia* L.	Nova Scotia, Florida Keys, South Dakota, Idaho, Nebraska, Oregon, California, Bermuda, Bahamas; the British Isles, Europe, North Africa, Lebanon.^[^ ^42^ ^]^	Kingdom: Planate Subkingdom: Spermatopsida Phylum: Tracheophytina Sub phylum: Euphyllophytina Infra phylum: Radiatopses Class: Spermatopsida Subclass: Aridae Super Order: Typhanae Order: Polaes Family: Typhaceae Genus: Typha angustata^[^ ^52^ ^]^	1.5–2.5 m^[^ ^42^ ^]^)	Dark green, sometimes reddish^[^ ^42^ ^]^	7–13^[^ ^42^ ^]^	3–15 mm^[^ ^42^ ^]^
			1.5–2 m^[^ ^52^ ^]^			5–12 mm^[^ ^52^ ^]^
*T. domingensis* Pers.	Oregon, California, Utah, Nevada, Arizona, Gulf States, Kansas, southern Florida; Atlantic coast, Maine; the Bahamas; West Indies, Central and South America, Argentina, Patagonia, the Philippines, New Guinea, New Caledonia, Australia and Tasmania.^[^ ^42^ ^]^	Kingdom: Plantae Phylum: Anthophyta Class: Monocotyledoneae Order: Typhales Family: Typhaceae Genus: Typha D.^[^ ^53^ ^]^	2.5–4 m^[^ ^42^ ^]^	Flat above, yellow‐green or pale green^[^ ^42^ ^]^	6–9^[^ ^42^ ^]^	3–20 mm^[^ ^42^ ^]^

## 3 CO_2_ Sequestration of Cattail Plants

**Figure 2:**
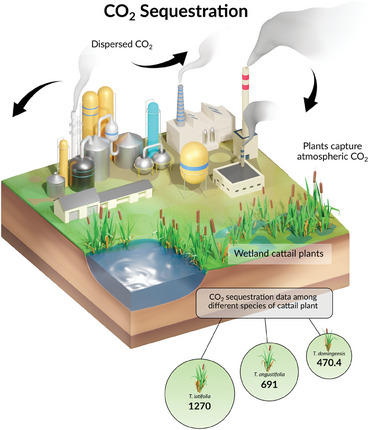
Schematic of Carbon dioxide sequestration by cattails. The sequestration data is in g CO_2_/Typha plant.

Cattails emerge as key participants in Carbon dioxide (CO_2_) sequestration efforts in the urgent quest to minimize the effects of climate change and restore ecological equilibrium. Using cattails to their full potential is essential for preventing climate change and promoting ecosystem resilience because they have a higher capacity to absorb and store CO_2_ than other conventional biomass fiber crops including flax, hemp, jute, sisal and kenaf. Carbon dioxide sequestration by cattail plants is shown schematically in **Figure** **2**. The data for CO_2_ sequestration of cattail plants among different species was determined in this study using the root biomass weight of the *Typha* plant. The data for the number of leaves, water depth, and weight of each dry cattail leaf were collected from published studies and the root biomass weight, weight of a Typha plant (with and without moisture content), and CO_2_ sequestration per Typha plant were calculated using Equations ((1), (2), (3), (4)), respectively. Irrespective of the *Typha* species' varieties, the moisture content percentage in *Typha* is kept constant during this calculation. The calculated CO_2_ sequestration data of various Typha plants, particularly the species *Typha latifolia*, *Typha angustifolia*, and *Typha domingensis* are summarized in **Table** **2**.

**Table 2:** CO_2_ sequestration data among different species of cattail plant.

Typha species	No. of leaves	Water depth [cm]	Weight of each dry leaf [g]	Root biomass weight [g]	Weight of a *Typha* plant [g]	Dry weight of a *Typha* plant [g]	Carbon content in *Typha*	Weight of carbon [g]	CO_2_ sequestration [g] per *Typha* plant
*T. latifolia*	14^a^ ^)^	50^c^ ^)^	34.1^c^ ^)^	35.33^c^ ^)^	512.7	461	75%*	346	1270**
*T. angustifolia*	10^a^ ^)^	50^c^ ^)^	25.9^c^ ^)^	19.94^c^ ^)^	278.9	251. 1		188.3	691**
*T. domingensis*	7.5^b^ ^)^	58^d^ ^)^	21.1^b^ ^)^	31.65^d^ ^)^	189.9	170.9		128.2	470.4**

^a)^ ^[^ ^42^ ^]^;

^b)^ ^[^ ^54^ ^];^

^c)^ ^[^ ^55^ ^];^

^d)^ ^[^ ^56^ ^]^.

*Moisture content in cattail is considered 10% for all three *Typha* species.^[^ ^57^ ^]^

**The weight of CO_2_ is 44. The ratio of CO_2_ to C is 44/12 = 3.67

(1) $$\begin{array}{cc} \mathrm{Root}\;\mathrm{biomass}\;\mathrm{weight}\;\left(g\right) & =\mathrm{Weight}\;\mathrm{of}\;\mathrm{each}\;\mathrm{dry}\;\mathrm{leaf}\;\left(g\right)\\ & \;\times \mathrm{No}.\;\mathrm{of}\;\mathrm{leaves}\;\times \;7.4\%\;(\mathrm{ramet}) \end{array}$$

(2) $$\begin{array}{cc} \mathrm{Weight}\;\mathrm{of}\;a\;Typha\;\mathrm{plant}\;\left(g\right) & =[\mathrm{No}.\;\mathrm{of}\;\mathrm{leaves}\;x\;\mathrm{Weight}\;\mathrm{of}\;\mathrm{each}\\ & \;\mathrm{dry}\;\mathrm{leaf}\;\left(g\right)]\\ & \;+\mathrm{Root}\;\mathrm{biomass}\;\mathrm{weight}\;\left(g\right)\; \end{array}$$

(3) $$\begin{array}{cc} \mathrm{Dry}\;\mathrm{weight}\;\mathrm{of}\;\mathrm{one}\;Typha\;\mathrm{plant}\;\left(g\right) & =\mathrm{Wt}\;\mathrm{of}\;a\;\mathrm{Typha}\;\mathrm{plant}\;\left(g\right)\\ & \;-[\mathrm{Wt}\;\mathrm{of}\;a\;\mathrm{Typha}\;\mathrm{plant}\;\left(g\right)\\ & \;\times \;\mathrm{moisture}\;\%] \end{array}$$

(4) $$\begin{array}{cc} & {\mathrm{CO}}_{2}\;\mathrm{sequestration}\;\left(g\right)\;\mathrm{per}\;\mathrm{Typha}\;\mathrm{plant}\\ & \;=\mathrm{Dry}\;\mathrm{wt}.\;\mathrm{of}\;\mathrm{one}\;\mathrm{cattail}\;\mathrm{plant}\;\left(g\right)\\ & \;\times \;\mathrm{carbon}\;\mathrm{content}\;\mathrm{in}\;\mathrm{cattail}\;\%\times 3.67 \end{array}$$

*T. latifolia* exhibited highest (≈1270 g CO_2_/*Typha*) carbon dioxide sequestration value and the *T. domingensis* exhibited the least value (≈470.4 g CO_2_/*Typha*) while the species *T. angustifolia* lies in between (≈691 g CO_2_/*Typha*). The higher carbon dioxide sequestration value of *T. latifolia* could be attributed to the greater leaf count and higher biomass, which collectively contribute to the overall increase in carbon uptake. However, a comprehensive comparison among various *Typha* species is challenging because of the distinctive characteristics that differentiate each species. For every 10 tons of dried cattail product, cattails absorb ≈1.47 tons of CO_2_ from the atmosphere to assimilate ≈4 tons of carbon.^[^ ^28^ ^]^ On the other hand, traditional biomass plant fiber, such as flax and hemp are grown in agricultural environments for producing fibers, oils, and textiles rather than C‐storage. Moreover, flax and hemp are annual plants and harvested annually, which must be replanted each year. Cattails, however, are perennial plants that grows in wetlands without requiring any agricultural activities and multiple harvesting is possible within a single growth cycle,^[^ ^57^, ^58^ ^]^ which validates the potential of *Typha* as a natural carbon sink over traditional biomass fiber crops.

## 4 Extraction of Cattail Fibers

The leaves, spikes, seeds, and stalks of several *Typha* species, including *T. Latifolia*, *T. Angustifolia*, and *T. Australis* can be used for extracting fibers.^[^ ^5^, ^37^, ^38^, ^40^, ^59^ ^]^ Cattail fibers are typically extracted using water retting, chemical retting, and mechanical decortication method.^[^ ^42^, ^45^ ^]^ Previous studies have explored different extraction methods to attain optimal fiber properties tailored for specific applications across various sectors. The retting conditions (e.g., concentration, temperature, and time) and methods (e.g., water retting, chemical retting, and mechanical decortication) chosen for fiber processing have a significant impact on the yield % of the extracted fibers. The extraction parameters and the yield % of cattail fibers obtained using various extraction methods are listed in **Table** **3**. Room temperature water retting process is cost‐effective; however, it requires longer retting duration (≈7 – 15 days) and yields lowest fiber percentage (≈0% – 9%). On the contrary, alkali retting of cattail fibers can be performed at varying times (≈2 – 10 h), temperatures (≈80 – 100 °C), and concentrations (≈2% – 10%), providing flexibility in design considerations while achieving maximum yield (≈8% – 47%). During water retting, pectinolytic bacteria attacks the pectin content in plant materials, which causes fiber separation after retting. Cattail leaves are reported to have no or less pectin content,^[^ ^5^ ^]^ while investigating for their chemical composition. Hence, the lack of active sites for microorganisms during the degumming process could be one of the reasons for the reduced effectiveness of water retting when exposed to cattail leaves.

**Table 3:** Retting conditions and yield % of cattail fibers obtained using various extraction methods.

Extraction process	Concentration [%], [g/l]	Temperature [°C]	Time	Fiber yield [%]	Reference
Water retting	–	20 °C	15 days	8.9	^[^ ^40^ ^]^
Water retting	–	20 °C	7 days	–	[60]
NaOH or CHзCOOH or Enzyme	3%	20 °C	16 days	No fiber	[11]
KOH	3%	80 °C	2 h	44.5	[11]
LiOH	3%	80 °C	2 h	46.6	[11]
NaOH	20 g l^−1^	100 °C	3 h	37.8	[61]
NaOH	7%	90 °C	10 h	34.5 ± 0.8	[38]
NaOH	5%	100 °C	2 h	32.3	[5]
KOH	5%	90 °C	4 h	8 – 30	[30]
NaOH	10–30 g l^−1^	0 – 100 °C	2‐4 h	25.1 – 46.6	[21]
NaOH	2%	Microwave	7 min	32.9 ± 2.8	[22]

Fiber yield of different *Typha* species is summarized in **Table** **4**. The amount of fiber per *Typha* plant is determined using Equation (5).

(5) $$T_{F}=D_{L}\;\times N_{L}\;\times \;Y$$

where, T_F_ is the amount fiber in g per *Typha* plant, D_L_ is the weight of the dry leaf in g, N_L_ is the average number of leaves per *Typha* plant, and Y is the average fiber yield. The data for the mass of the dry leaf and number of leaves per *Typha* plant is collected from the literatures to calculate T_F_. *Typha* species are ranked as highest and lowest based on the amount of fiber data calculated for different species. The highest amount of fiber was obtained from *T. latifolia* (≈190.9 g) and the lowest from *T. Australis* (≈59.7 g), while the species *T. Angustifolia* and *T. domingensis* lying in between. *T. latifolia* and *T. Australis*, despite having similar numbers of leaves per plant, the former stands in the upper extreme and the latter stands in the lower extreme of the chart (Table 4). This is because of the greater average width of the leaves for *T. latifolia*, resulting in a higher mass of the dry leaves and the total amount of fiber. Given that variations in *Typha* species result in variances in the fiber obtained from each plant due to differences in leaf height, breadth, and weight, consistency in extraction medium is required for an impartial comparison. Hence, further study is required for more accurate and in‐depth discussion on this.

**Table 4:** Ranking of various Typha species based on the fiber yield per plant.

Typha species	Weight of the dry leaf [g]	Average number of leaves per plant	Dry weight of a plant [g]	Average fiber yield [%]	Amount of fiber [g] per *Typha* plant	Ranking
*T. latifolia L*.	34.1^a^	14^d^ ^)^	477.4	40^e^ ^)^	190.9	★★★★
*T. Angustifolia*	25.9^a^ ^)^	10^d^ ^)^	259	70^f^ ^)^	181.3	★★★
*T. domingensis*	21.1^b^ ^)^	7.5^b^ ^)^	158.3	41.6^g^ ^)^	65.8	★★
*Typha Australis*	13.2^c^ ^)^	14^c^ ^)^	184.8	32.3^h^ ^)^	59.7	★

^a)^ ^[^ ^55^ ^]^;

^b)^ ^[^ ^54^ ^]^;

^c)^ ^[^ ^62^ ^]^;

^d)^ ^[^ ^42^ ^]^;

^e)^ ^[^ ^11^ ^]^;

^f)^ ^[^ ^63^ ^]^;

^g)^ ^[^ ^64^ ^]^;

^h)^ ^[^ ^5^ ^]^.

Note: ★★★★ – highest; ★ – lowest

### 4.1 Alkaline Degumming

**Figure 3:**
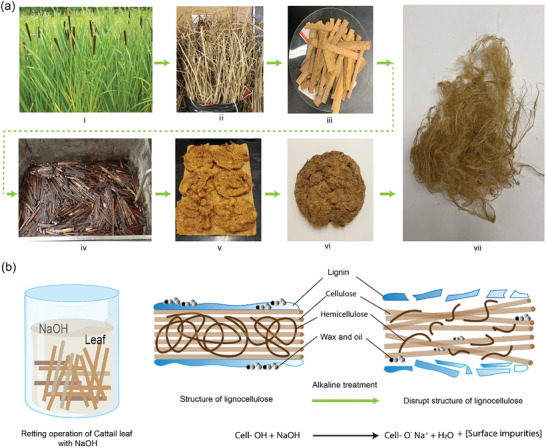
a) Conversion of cattail plants into lignocellulosic fibers (adapted.^[^ ^29^ ^]^ unrestricted permission) i) Cattail plants in wetland. ii) Collected cattail leaves stored in laboratory. iii) Precut cattail leaves for alkali retting. iv) Ongoing retting operation of cattail with KOH. v) Neutralized and washed cattail fiber after retting. vi) Dried (at room temperature) cattail fiber bundle. vii) Individualized cattail fibers. b) schematic of the mechanism of retting operation of cattail leaf with NaOH.

Alkaline degumming is the most widely investigated methods for obtaining cattail fibers, which involves treating the precut cattail plants at a given retting temperature and time in aqueous alkaline solution followed by rinsing, washing, and drying (**Figure** **3**a). Cattail fibers were extracted before^[^ ^11^ ^]^ for textile applications from *Typha latifolia* leaves and the core spongy stem tissue by varying the type of alkaline solutions, treatment temperature and time. Fiber yield was reported to be ≈30%–50% in this study, with a decrease in fiber yield as extraction temperatures and times increased. Following this, cattail fibers from leaves using NaOH solution and optimized the extraction parameters, i.e., time, temperature, and concentration for composite applications was extracted in a study,^[^ ^38^ ^]^ using desirability function analysis (DFA), where the desirability functions are given by Equations ((6), (7), (8)) for maximizing a property (*Yi*) and Equations ((9), (10), (11)) for minimizing a property (*Yi*). Here, C corresponds to the value of upper criteria when maximizing and lower criteria when minimizing, *Y_min_* and *Y_max_* are the value of upper and lower tolerance, respectively, and s indicates the weight. This study concluded 7%, 10 h, and 90 °C as optimal treatment concentration, duration, and temperature, respectively, and a fiber yield of ≈34.5% under these specified retting conditions. However, adjustments in the range, weight, and emphasis on the coefficient of individual responses can cause alteration of the optimal conditions. In addition, a chemical degumming process was employed^[^ ^65^ ^]^ to extract the *Typha* fibers using aqueous NaOH and urea solution, with urea acting as an auxiliary agent. This study reported 15 g L^−1^ as the optimal NaOH concentration and 5 g L^−1^ as the optimal urea concentration for extracting cattail fibers at 95 °C for 3 h. The mechanism of alkaline degumming of cattail fibers is illustrated in Figure 3b. Like other lignocellulosic plant fibers, alkaline degumming modifies the surface structure of cattail fibers by removing low‐molecular weight components and breaking up the outer fiber cell wall surface shielded with gummy materials. This consequently results in a rugged surface topography and increases the cellulose % and reduces the fiber diameter.

(6) $$d_{i}=0\;\mathrm{if}\;Y_{i}\;<\;Y_{min}$$

(7)

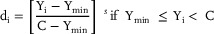

(8) $$d_{i}=1\;\mathrm{if}\;Y_{i}\;\geq \;C$$

(9) $$d_{i}=1\;\mathrm{if}\;Y_{i}\;\leq \;C$$

(10)

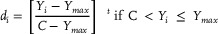

(11) $$d_{i}=0\;\mathrm{if}\;Y_{i}\;>\;Y_{max}$$

### 4.2 Water Retting and Other Methods

Apart from the alkaline degumming, extraction of *Typha* fibers have also been investigated using manual decortications, sea water retting (FM) and a combination of alkaline (NaOH) and enzymatic (Pectinex Ultra SPL) degumming processes.^[^ ^16^ ^]^ While seawater retting is a more sustainable approach, it took much longer (4 weeks) to accomplish retting when compared with manual decortications and chemical retting. However, sea water retted *Typha* fibers exhibited higher crystallinity index (CI) (∼42.6%) than those of manual decorticated fibers (≈40.1%) and comparable CI with those of chemically retted fibers (≈49%–63%). Cattail fibers were also explored for extraction via water retting, wherein crushed leaves were bundled and submerged in a water tank for 15 days, and subsequently covered with hay.^[^ ^40^ ^]^ The fiber yield of the extracted cattail fibers in this study was ≈8.95%, which is lower than that of the fiber yield obtained using chemical retting. This is believed to be due to the removal of the non‐cellulosic components from the plant materials when dissolved in chemical reagents, whereas water retting solely relies on microbial action to break the bonds between the fibrous and non‐fibrous parts. Although Typha fibers were successfully extracted using sea water^[^ ^16^ ^]^ and tap water,^[^ ^40^ ^]^ attempts using distilled water at room temperature (21 °C) for 90 days and at an elevated temperature (80 °C) for 6 h were unsuccessful in extracting fibers from *Typha* plants.^[^ ^11^ ^]^ Hence, further investigation is required to rule out fiber extraction and processing, concomitantly aiming to mitigate retting duration.

The most recent advancement in cattail fiber extraction involves the utilization of the microwave radiation volumetric method using alkaline solution instead of conventional heating bath.^[^ ^22^ ^]^ Microwave retting utilizes the electromagnetic spectrum, slashing retting time from 2 to 10 h for chemical retting and 7 to 28 days for water retting down to just 7 min. The fiber yield obtained during microwave retting is ≈26% to 34%, which is comparable to that of chemical retting.^[^ ^22^ ^]^

### 4.3 Factors Affecting Fiber Properties

It is evident that the extraction conditions, such as temperature, time, and the concentration of the solvent employed during retting, determine the fiber properties (e.g., fiber yield and mechanical characteristics). A delicate balance of these parameters is required to achieve optimal fiber properties. For instance, a maximum fiber yield was achieved at lower retting temperature and time.^[^ ^11^ ^]^ However, maximum fiber yield at lower retting temperature and concentration with prolonged retting time was achieved in a study;^[^ ^38^ ^]^ nonetheless, the mechanical properties of the fibers increased with the increase in both temperature and time. These variations are believed to be due to the variations in plant maturity and growth within and between different plants and the variations in the interaction intensity of the independent parameters at different levels. In addition to extraction methods, the location of Typha leaves from which fibers are extracted can affect fiber processing and resultant properties.^[^ ^66^ ^]^ When divided into distinct zones, such as top, middle, and bottom, the mechanical properties of cattail fiber demonstrated sensitivity to leaf locations, with the middle zone exhibiting the highest tensile strength (≈103.5 MPa) and modulus (≈5.6 GPa) compared to fibers from the bottom and top zones. Furthermore, the yield % of the cattail fibers varied substantially when investigated among different growth stages of cattail leaves, with a minimum yield (≈7.4%) obtained for the late flower (LF) stage and a maximum yield (≈28.9%) obtained for the mature stage.^[^ ^57^ ^]^ The differences in the fiber yield were reported to be attributed to the substantial variations in water content of the cattail plants at different growth stages (late flower: ≈78%; mature: ≈10.4%).^[^ ^57^ ^]^

### 4.4 Comparison Among Various Extraction Methods

Water retting is a sustainable and cost‐effective method for extracting cattail fibers. However, water retting consumes way too long time and causes lower fiber yield compared to chemical degumming methods. Similar to water retting, manual extraction method demonstrates low efficiency and fiber yield. Although the mechanical decortication process can increase retting efficiency and fiber yield, it requires a large‐scale investment. Furthermore, limited information on the mechanical decortication processes of the cattail fibers is available. Microwave retting exhibits higher fiber yield with the shortest retting time; however, it requires a large material‐to‐liquor ratio (1:60)^[^ ^22^ ^]^ when compared with alkali retting (1:20). Furthermore, microwave retting is not yet investigated for scalable fiber production. Chemical retting techniques, on the other hand, exhibit higher efficiency and fiber yield while simultaneously reducing retting time and enhancing fiber refinement. Among various chemical retting, alkaline degumming emerges as the most straightforward, cost‐effective, and efficient method, yielding more uniform fibers. The use of chemicals renders it a less environmentally friendly approach. However, the alkaline solution used during retting can be recycled for subsequent fiber extraction up to 17 times, with fiber properties statistically insignificant for up to 14 cycles.^[^ ^67^ ^]^

## 5 Characterization of *Typha*

### 5.1 Leaf Structure and Morphology

Traditional bast fibers are characterized by the selective conversion of the exterior stem tissue into fibrous constituents, contrasting with other structural elements of the plant. In contrast to conventional bast fiber sources, the cattail plant presents a unique attribute whereby the entire leaf structure undergoes transformation into fibrous material, diverging from the conventional paradigm of bast fiber development. This is why cattail fibers exhibit higher yield, lower density, and higher mechanical properties. Hence, it is therefore necessary to understand the structure of the cattail leaf, which determines the fiber structure and properties.

**Figure 4:**
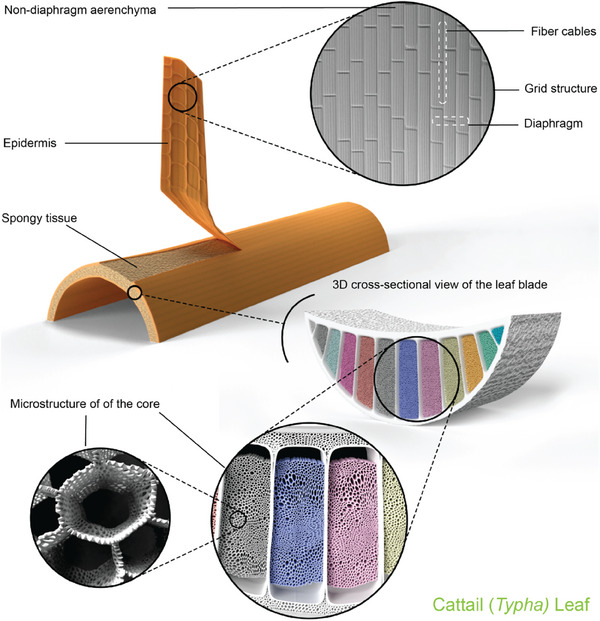
Macro and micro structural model of cattail leaf.

A schematic illustration of the macro and microstructural model for cattail leaf is shown in **Figure** **4**. The Typha leaf consists of a sandwich structure having bark or epidermis and spongy tissue. The spongy tissues form the core structure of the leaf blade. The thickness of this spongy core follows a quadratic curve, increasing in successive layers from the outer leaf toward the plant's center.^[^ ^11^ ^]^ At micro level, cattail leaf shows diaphragm, partition, fiber cables, hollow and a porous structure.^[^ ^68^, ^69^, ^70^ ^]^

The longitudinal dorsal view of the leaf blade (Figure 4) consists of the non‐diaphragm aerenchyma, fiber cables, partitions, and diaphragms. Diaphragms are composed of multiple layers of thin‐walled aerenchyma tissue. Diaphragms run parallel to each other and perpendicular to the partition, intersected by fiber cables that form a grid structure.^[^ ^70^, ^71^, ^72^ ^]^ The parallel diaphragms connect and hold the ventral and dorsal surfaces of the epidermis, and enhance the flexural properties, and allow leaves to float in water by facilitating fluid exchange.^[^ ^69^, ^70^, ^73^ ^]^ Furthermore, the overall grid structural model of the epidermal surface of the cattail leaf exhibits superior energy‐absorbing capabilities under axial compression and lateral bending compared to the typical thin hollow tube model.^[^ ^70^ ^]^ The microstructures of the spongy tissues (Figure 4) are established with a network predominantly distributed in the regions distal to the epidermis.^[^ ^70^, ^74^ ^]^ The core structure, which consists of spongy tissues, is predominantly hollow and porous, contributing to the lightweight nature of cattail fibers.

### 5.2 Fiber Structure and Morphology

**Figure 5:**
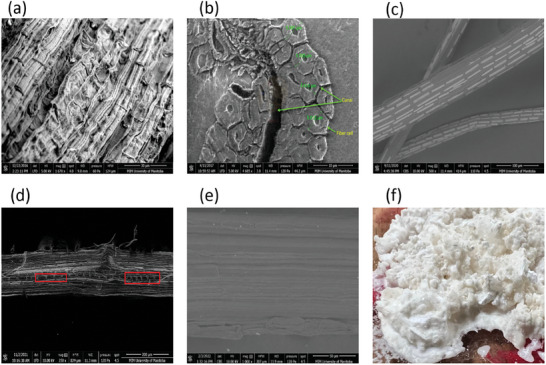
Structures of cattail fibers. SEM image of – a) longitudinal view of cattail fiber bundle b) cross‐sectional view of cattail fibers; (a,b: reproduced with permission.^[^ ^11^ ^]^ Copyright 2021, The Korean Fiber Society) c) longitudinal view of cattail fibers showing calcium oxalate plates (COPs) (source: ^[^ ^77^ ^]^ unrestricted permission) d) individual cattail fiber showing pit areas (source^[^ ^3^ ^]^ unrestricted permission). e) complete removal of COPs after ultrasonic cleaning f) left solution after ultrasonic cleaning for COPs removal.^[^ ^22^ ^]^

The SEM image for a longitudinal and cross‐sectional view of cattail fiber bundles, extracted using cattail leaves and alkaline retting, are shown in **Figure** **5**a,b, respectively. The cross‐section of the fiber consists of elliptical cells and irregularly spaced and structured canals and lumens. Unlike flax and hemp, cattail fibers have a relatively narrower lumen and a rough surface with multiple ridges connected by a crenellated structure.^[^ ^29^ ^]^ The cattail leaf fiber exhibits a unique surface structure, which is made up of irregularly spaced rectangular calcium oxalate plates (COPs) of different lengths and widths across different locations (Figure 5c) and excavated or pit areas (Figure 5d). The presence of COPs and pit regions may be seen in cattail leaf fibers, irrespective of the extraction techniques employed.^[^ ^16^, ^22^, ^50^ ^]^ These COPs originate from the outer layer of the fiber cables, containing prismatic calcium oxalate crystals, which were confirmed while investigating the crystals on the fiber cables isolated from the leaves.^[^ ^71^, ^75^, ^76^ ^]^

Researchers have attempted to remove COPs from cattail fibers using ultrasonic cleaning and ethylenediaminetetraacetic acid (EDTA).^[^ ^22^ ^]^ The surface image of cattail fibers after removal of COPs and the crystals obtained from the resultant left‐over chemical solution generated after ultrasonic cleaning are shown in Figure 5e,f, respectively. The removal of the COPs from the cattail fiber surface doesn't cause statistical changes in the tensile strength and modulus of fibers. However, it does lead to a reduction in fiber density of ≈10.9%. Given the fiber properties are the same before and after COPs removal, the reduction in fiber density after COPs removal would result in an increase in specific properties (e.g., specific strength and specific modulus). This consequently will result in lighter‐weight composite parts manufactured from cattail fibers with higher specific properties. Hence, the surface engineering of cattail fibers should be considered as a key aspect for bridging the knowledge between materials properties and product design and manufacturing.

### 5.3 Chemical Composition

**Figure 6:**
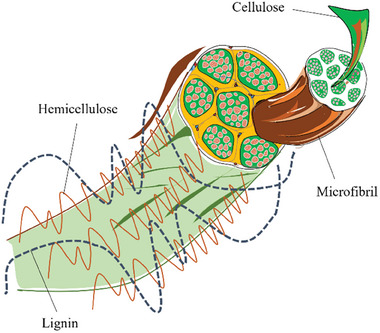
Schematic illustration of chemical components and microfibrils cross‐section in plant fiber cell wall structure (reproduced with permission from ^[^ ^29^ ^]^ © 2023 Elsevier B.V.).

Cattail is a source of lignocellulosic fibers^[^ ^21^, ^45^ ^]^ of which cellulose, hemi‐cellulose, lignin, pectin, and other water‐soluble materials are the principal chemical constituents.^[^ ^78^ ^]^ The chemical components and microfibrils cross‐section in plant fiber cell wall structure is shown schematically in **Figure** **6**. Most non‐cellulosic materials are removed from *Typha* fibers during their extraction process using alkaline chemicals.^[^ ^5^ ^]^ This result in a decrease in the amount of hemicellulose and non‐cellulosic materials and an increase in the cellulose content. Since cellulose has a high tensile strength and crystallinity, it has the greatest effect on the physical characteristics of fibers. While closer to that of jute, sisal, and oil palm fibers, the cellulose content of *Typha* fibers is higher than that of kenaf and coir fibers. **Table** **5** represents the chemical composition of the cattail fiber investigated in different studies.

**Table 5:** Chemical composition of various species of cattail fibers.

Species	Fiber type	Cellulose [%]	Hemi‐cellulose [%]	Lignin [%]	Wax [%]	Pectin [%]	Ash [%]	References
*T. latifolia L*.	Spikes	22.4 ± 0.7	21.8 ± 0.5	20.6 ± 0.5	11.5 ± 0.1	0.18 ± 0.2	4.5 ± 0.1	[79]
*T. domingensis*	Leaves	68.35	–	17.6	0.37	–	5.71	[40]
*T. domingensis*	Seeds	73.46	–	9.88	0.93	–	5.9	[40]
*T. latifolia L*.	Dried Leaves	55‐65	8 – 10	8 – 10	–	–	4 – 8	[11]
*T. australis*	Leaves	47.4	14.4	28.6	–	–	–	[5]
*T. australis*	Powdered leaves	43.5 ± 3.5	22.1 ± 2.7	32.5 ± 1.5	–	–	–	[5]
*T. angustifolia*	Leaves	68.3	7.3		–	–	–	[5]
*Tunisian Typha*	Leaves	67.3	–	13.7	–	–	–	[5]

**Figure 7:**
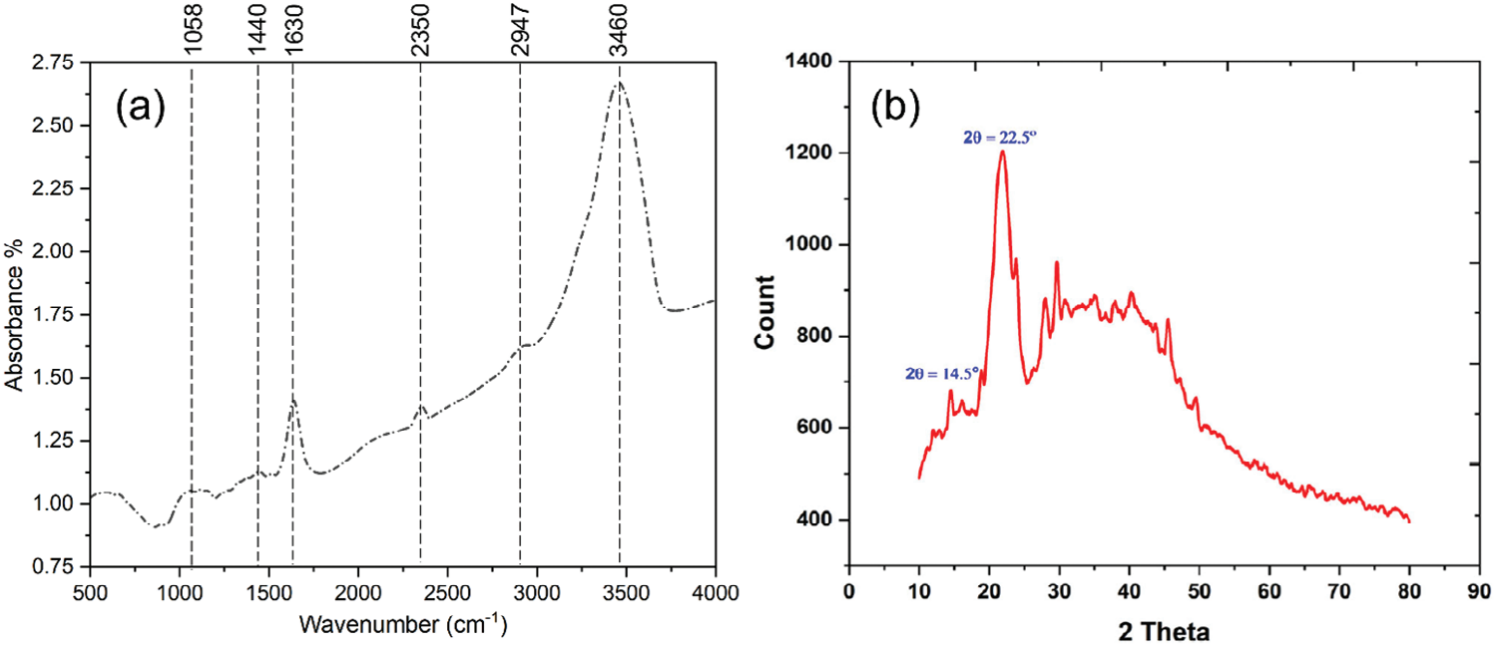
Characterization of cattail fibers. a) FTIR spectra of Typha fiber. b) XRD spectra of Typha fiber (b: reproduced with permission.^[^ ^60^ ^]^ © 2023 Society of Plastics Engineers).

The FTIR spectra of cattail fiber extracted from cattail leaves is illustrated in **Figure** **7**a. The cattail fibers exhibited peaks at ≈1058, 1630, 2947, and 3460 cm^−1^ which is associated with the vibration of C─OH stretching of the cellulose backbone, absorbed water, C─H stretching in cellulose and hemicellulose, H‐bonded OH groups in cellulose, respectively.^[^ ^29^ ^]^ The reduction in lignin components is observed in cattail leaves when compared with cattail leaves^[^ ^11^ ^]^which is believed to be due to fiber refinement after alkaline retting. The waxiness of cattail fiber proved to be very high due to the stretching vibration of the ─OH group which is the reason for the broader peak at 3363 cm^−1^. Cattail fiber has 10.64% waxiness which is much higher than kapok and cotton fiber.^[^ ^80^ ^]^ X‐ray diffraction of cattail fiber is shown in Figure 7b. Cattail fibers exhibited diffraction peaks at ≈14.5 and 22.5°, which corresponds to the crystalline cellulose and amorphous component of the plant materials, respectively.^[^ ^60^ ^]^

### 5.4 Physical Properties

**Figure 8:**
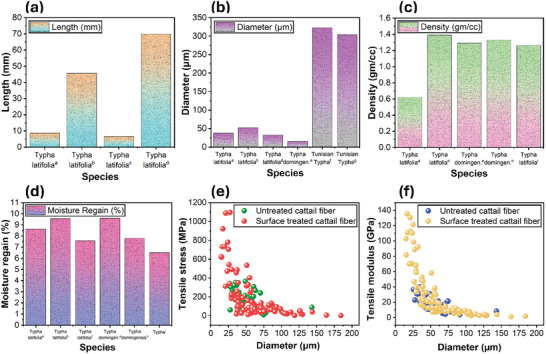
Physical properties of different species of Typha fiber; a) fiber length b) fiber diameter c) fiber density d) moisture regain (a–d: data collected.^[^ ^5^, ^21^, ^30^, ^37^, ^40^, ^61^, ^79^, ^86^ ^]^). e) variations in tensile strength of untreated and surface treated cattail fiber. f) variations in tensile modulus of untreated and surface‐treated cattail fiber (e,f: data collected from^[^ ^85^ ^]^).

The physical properties, fiber length, fiber diameter, density, and moisture regain, of different *Typha* species are shown in **Figure** **8**a–d, respectively. Engineering these fiber features is crucial to convert cattail biomass into value added bioproducts. For instance, finer fiber diameter facilitates yarn spinning, lower fiber density improves specific properties of composites, reduced fiber moisture content enhances compatibility with polymer matrices, and so forth. The length of cattail fibers obtained after extraction varied between ≈8 and 70 mm (Figure 8a). The variation in cattail fiber length is primarily attributed to factors such as extraction method (e.g., water retting, chemical retting), fiber source (e.g., leaf, seed, spike), and precut length prior to extraction, in addition to variations in fiber length among different species.^[^ ^11^, ^30^, ^37^, ^79^ ^]^

Like other natural fibers, the fibers extracted from different *Typha* species exhibited a large distribution and varied between ≈10 and 500 µm (Figure 8b). Apart from the variations in the *Typha* species, the distribution in the fiber diameter is attributed to the variations in retting conditions, i.e., retting time, temperature, and concentration^[^ ^38^ ^]^ and heterogeneity in fiber structures.^[^ ^3^ ^]^ These factors, together with the variations in fiber source (e.g., leaf, seed, spike), contribute to the variations in fiber density (≈0.62–1.39 g cm^3^) as shown in Figure 8c. Cattail fiber is lighter than flax (≈1.49 g cm^3^)^[^ ^81^ ^]^ and hemp (1.57 g cm^3^)^[^ ^82^ ^]^ fibers, which is believed to be due to its hollow structure.

The amount of water absorbed by the fibers is usually given by % of moisture regain. For apparel applications, moisture regain is used to adjust the water content during commercial transactions, however, for industrial applications of fibers, moisture content instead of regain value is used in line with other biological materials. The moisture regain values of different species of cattail are shown in Figure 8d. According to past study,^[^ ^3^ ^]^ cattail fiber exhibit ≈15% moisture content (MC) at 65% relative humidity (RH), which is higher than those of flax (≈7%), hemp (≈9%), jute (≈12%), and sisal (≈11%) fibers tested at the same RH.^[^ ^83^ ^]^ However, further increase in MC was observed with increase in RH until it reaches the equilibrium value, mirroring trends observed in cotton, flax, hemp, jute, and sisal fibers.^[^ ^84^ ^]^ Cattail fiber exhibits 72% to 79% dye exhaustion, which is in the range of dye exhaustion of cotton fiber (≈50%–90%), while the color fastness value also meets minimum ASTM performance standards.^[^ ^11^ ^]^

### 5.5 Mechanical Properties

The tensile strength of cattail fibers varies between ≈10 and 360 MPa and the tensile modulus between ≈3 and 40 GPa. The variability in the mechanical properties of cattail fiber is primarily attributed to variations in fiber diameter, which typically vary within the range of 25 to 145 µm.^[^ ^85^ ^]^ The tensile strength and modulus typically increase with a decrease in cattail fiber diameter, as illustrated in Figure 8e,f, respectively, consistent with the trend observed in other lignocellulosic fibers. The mechanical properties of cattail fibers can be further engineered by tailoring their surface with chemicals,^[^ ^85^ ^]^ leading to an increase in both tensile strength (from ≈105 to ≈303 MPa; mean value) and tensile modulus (from ≈9.8 to ≈38.7 GPa; mean value) after surface treatment. The efficacy of surface treatment has been demonstrated only within a chemical reagent concentration range of 5% to 10%, while the cattail fibers treated at 2.5% concentration exhibit properties within the scatter range of untreated fibers (Figure 8e,f).

## 6 Applications

### 6.1 Fiber Reinforced Composites

Sustainable fiber reinforced polymer (FRP) composites from renewable and biodegradable fibrous materials and polymer matrices are of great interest, as they can potentially reduce environmental impacts.^[^ ^87^, ^88^ ^]^ However, the overall properties of such composites are still far from the high‐performance conventional glass or carbon FRP composites. Therefore, a balance between composite performance and biodegradability is required with approaches to what one might call an eco‐friendly composite. Composites manufactured with biobased fibers as reinforcement have gained enormous attention in recent decades, driven by their increasing demand in aerospace, automotive, construction, and agriculture industries as substitutes for synthetic fibers like aramid, carbon, and glass. These green materials provide sufficient stiffness and strength while remaining cost‐effective, making them attractive options for sustainable composite products.^[^ ^1^, ^81^, ^89^, ^90^, ^91^, ^92^ ^]^ Polymer matrix composites (PMC) are made up of reinforcing element and polymeric matrix, and biocomposites are created by combining biologically produced components with one or more fiber type.^[^ ^93^, ^94^, ^95^ ^]^ High‐strength natural fibers, such as jute, oil palm, sisal, kenaf, and flax are embedded in a polymer matrix to form natural fiber‐reinforced polymer composites (NFRCs).^[^ ^91^ ^]^ However, the low yield (5%–15%), poor water resistance, low durability, and poor fiber‐matrix interfacial bonding of natural biomass fibers, like flax, hemp, jute, sisal, kenaf, coir, kapok and banana reduces the final properties of the composites and limit their industrial applications.^[^ ^29^, ^38^ ^]^ Moreover, searching for sustainable raw materials with superior qualities is cumbersome because most natural fibers are limited to specific geographical areas.^[^ ^96^ ^]^

**Figure 9:**
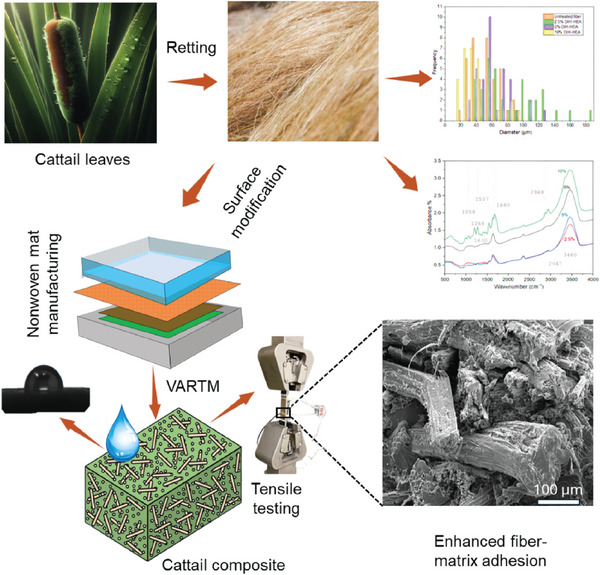
Cattail composites manufactured from modified surface cattail fibers (Data adapted from^[^ ^85^ ^]^).

*Typha Latifolia* is one of the wetland plants being investigated as a possible emerging renewable fiber source^[^ ^11^, ^29^, ^77^ ^]^ due to their availability, cellulose content in their chemical composition, abundance, feed value, medicinal properties, capacity to filter water, resilience to flooding, and ability to serve as wildlife habitat. Several studies have investigated the suitability of *Typha* fibers as reinforcement in composites.^[^ ^5^ ^]^ **Table** **6** below summarizes the list of methods that were employed by previous studies in manufacturing composites using cattail fiber together with their physical and mechanical properties and intended applications. Cattail fiber was performed in a study^[^ ^77^ ^]^ into the nonwoven mat and manufactured composites through Vacuum‐assisted resin transfer molding (VARTM) for applications in transportation industries. Subsequently, additional pressures were applied to investigate the effect of consolidation pressure on composite properties. As concluded by that study,^[^ ^77^ ^]^ the structure and design (i.e., thickness and areal density) of the nonwoven mat impacted their properties (e.g., fiber volume fraction and permeability of the mat), which together with the consolidation pressure during manufacturing affected the physical and mechanical properties of the cattail fiber composites. The longitudinal modulus of cattail composites varied between 4.6 and 7 GPa, and tensile strength between 18.6 and 44.1 MPa, which is comparable to the composite properties manufactured using flax, hemp, and hybrid flax‐hemp mats.^[^ ^30^, ^81^, ^82^ ^]^ Further extending this research,^[^ ^85^ ^]^ modified the surface of cattail fibers by employing DIH‐HEA treatment to enhance the performance of cattail fiber composites by improving interfacing bonding between the fibers and resin matrix. The manufacturing and characterization of the cattail mat composites manufactured using modified surface cattail fiber is shown schematically in **Figure** **9**.

**Table 6:** Overview of cattail fiber composites manufactured using various methods.

Composite Type	Manufacturing method	Resin used	Physical & mechanical properties	Application	References
Nonwoven mat composite	VARTM (Vacuum‐assisted resin transfer molding)	Unsaturated Polyester resin	Thickness: 2.2–6 mm	Automobile industries	[30]
			Density: 1.19–1.23 g cm^3^		
			Longitudinal modulus: 4.6–7 GPa		
			Tensile stress: 18.6–44.1 MPa		
			Strain at break 0.4%–1.0%		
Natural Fiber Reinforced Polymer Composites (NFRPC)	Hand layup method	Epoxy resin	Tensile Strength (short fibers): 1.3 MPa	Underwater transportation	[97]
			Tensile strength (long fibers): 4.24 MPa		
Novel binder‐free fiber‐reinforced composites	–	No Glue/Binder	Flexural modulus: 3100 ± 92 MPa	Automotive/ Furniture industries	[41]
			Flexural strength: 21 ± 2 MPa		
Novel composite aerogel adsorbent ZCCA	Dispersion method		Maximum adsorption capacity: 172.09 mg g^−1^	Water treatment industries	[98]
Hybrid‐reinforced polyester composites (Sisal/Cattail)	Hand layup method	Polyester	Flexural strength: 45.9 MPa	Light‐weight Composite Industries	[1]
			Tensile strength: 32.4 MPa		
*Typha angustifolia* fiber reinforced (TAFR) composite	Compressive method	Epoxy	Crystallinity index: 37.58%	Low to medium weight composites	[65]

**Figure 10:**
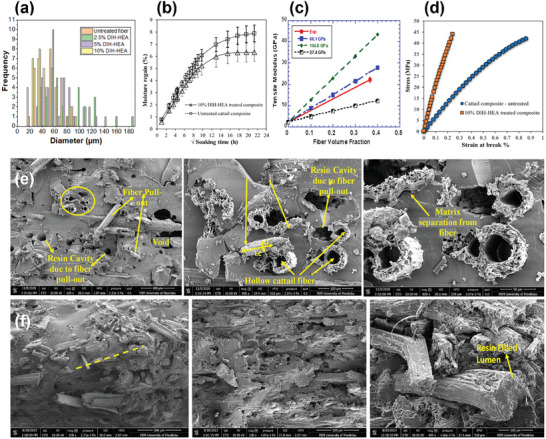
Composites by cattail. a) distribution in diameter of untreated cattail and treated fibers b) variation in water absorption behavior of untreated and treated cattail fiber composites c) predicted modulus for oriented continuous fiber composites with surface treated cattail fibers d) stress–strain behavior of untreated and treated cattail fiber composites. SEM images of the fractured surfaces of the‐ e) cattail fibers treated with 5% DIH‐HEA f) cattail fibers treated with 10% DIH‐HEA – at various magnifications (a–f: adapted from^[^ ^85^ ^]^ unrestricted permission).

Surface‐treated cattail fibers showed a significant reduction in diameter, as shown in **Figure** **10**a, ranging from 15 to 90 µm for those treated with 10% DIH‐HEA, with an average diameter of 34.8 µm, compared to untreated fibers, which had a diameter range of 22 to 143 µm and a mean of 55.3 µm (Figure 10a). This reduction in diameter contributed to an increase in both the modulus and strength of the fibers. Despite this, the untreated and surface‐treated cattail composites exhibited similar diffusion kinetics, likely due to the random orientation and discontinuous nature of the cattail fibers embedded in the matrix. However, the equilibrium water uptake of the cattail composites (M_m_) decreased by ≈19.5% from 7.88 to 6.34 when manufactured using the fibers treated with 10% DIH‐HEA (Figure 10b).

The changes in fiber properties after surface treatment have also been reflected in the experimental and predicted modulus (Figure 10c) and stress‐strain behavior (Figure 10d) of the treated cattail composites. A substantial difference in the stress‐strain behavior of composites manufactured with or without surface treatment was observed as shown in Figure 10d. As shown in Figure 10c, the predicted tensile modulus of cattail fiber composites, determined using the properties of surface treated fibers, is ≈66.1 and 3.42 GPa for [0] and [90] orientation, respectively. It is expected that the randomly oriented discontinuous fiber composites act as a quasi‐isotropic material and the longitudinal‐[0] and transverse‐[90] modulus of such material type appears to be same. However, the average experimental tensile modulus (≈22.2 GPa) of surface treated cattail fiber composites, as shown in Figure 10c,d, lies in between predicted longitudinal and transverse modulus, suggesting the preferential orientation of cattail fibers while being embedded in the matrix.

The enhancement in interfacial bonding between the fibers and matrix was also prominent due to surface treatment (Figure 10e,f). The enhancement in interfacial bonding was quantified by measuring the interfacial bond shear strength (IBSS, *τ_u_*). *τ_u_* increases from 16 MPa for untreated composites to 22 MPa for treated composites, which is believed to be due to the increase in the critical fiber length (*l_c_*) in the fractured composite surfaces (Figure 10e,f). Apart from these studies, cattail fibers have also been used together with sisal^[^ ^1^ ^]^ and bamboo fibers^[^ ^97^ ^]^ to manufactured hybrid natural fiber‐reinforced polymer composites (NFRPCs).

### 6.2 Sensors

Sensors are typically manufactured using conductive materials, such as graphene and carbon nanotubes and flexible materials, such as Ecoflex, polydimethylsiloxane (PDMS), and thermoplastic polyurethane elastomer (TPU).^[^ ^99^ ^]^ However, the sensors manufactured using these materials are not sustainable in their as‐prepared state and require a complicated and expensive fabrication process. Cattail fibers offer great potential as bio‐based flexible sensing materials for fabricating sensors, such as pressure sensors and gas sensors, due to their flexibility, cost‐effectiveness, eco‐friendliness, ease of preparation, and high sensitivity.

**Figure 11:**
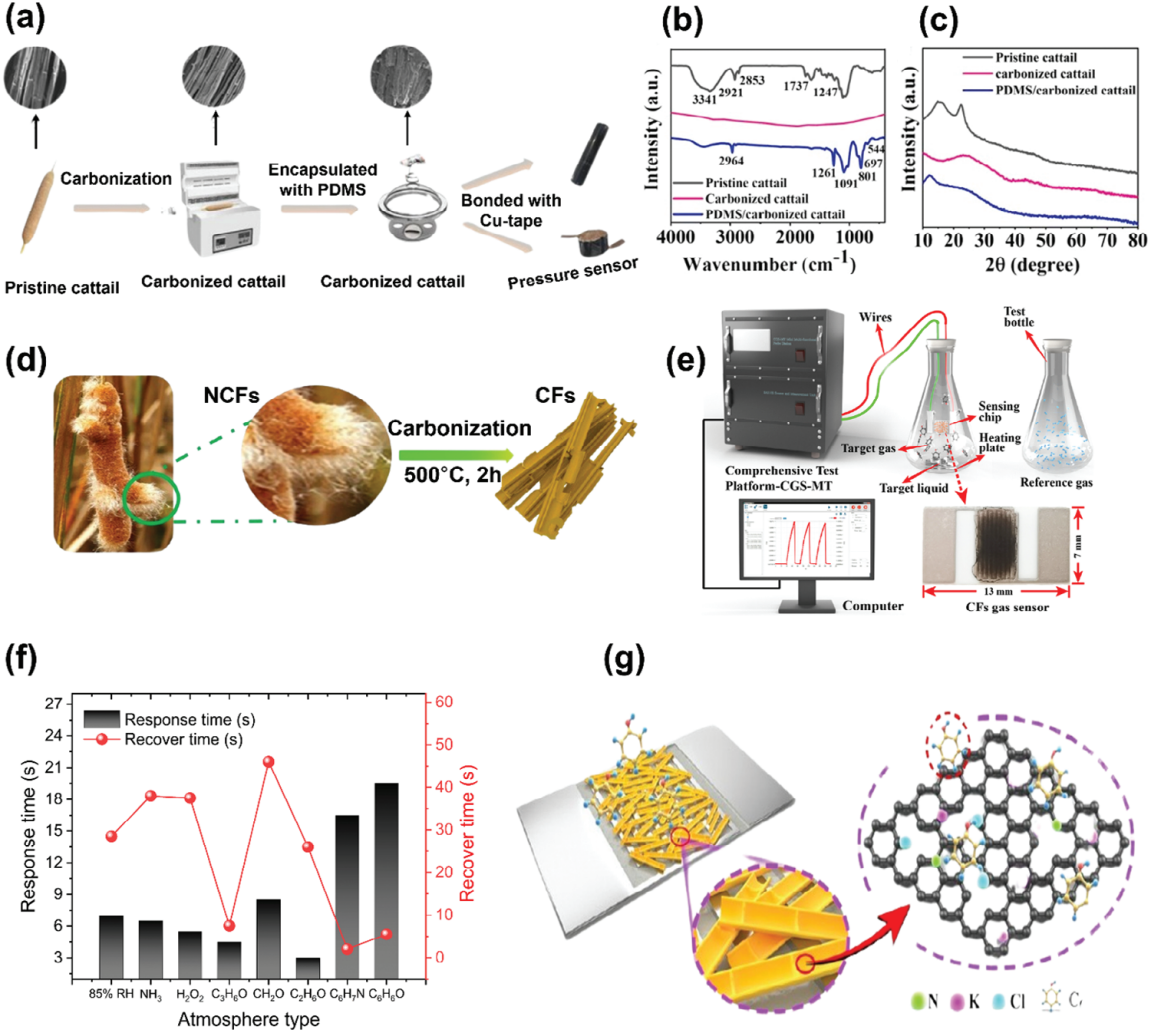
Sensor applications by cattail. a) Schematic illustration process for manufacturing carbonized cattail pressure sensor b) FTIR spectra of the cattail fibers before and after carbonization c) XRD spectra of the cattail fibers before and after carbonization (a–c: reproduced with permission.^[^ ^99^ ^]^ © 2023 Elsevier Ltd.); d) converting waste cattail into carbon fibers via carbonization e) characterization of cattail fiber based gas sensor; sensing performance cattail fiber based gas sensor f) response time and recovery time; g) mechanism of cattail fiber based gas sensor (d, e, g: reproduced with permission.^[^ ^102^ ^]^ © 2023 Elsevier Ltd.).

Cattail fiber has been investigated for manufacturing pressure sensors using carbonized cattail fiber (conductive element) encapsulated with polydimethylsiloxane (PDMS) (flexible substrate material) through vacuum negative pressure method (**Figure** **11**a).^[^ ^99^ ^]^

The structural integrity of cattail fiber remains unaltered after carbonization; however, carbonized cattail fibers exhibited ∼25.8%, ≈32.8%, and ≈54.7% reduction in fiber diameter, length, and weight, respectively, when compared with pristine fibers. The infiltration of PDMS into cattail fiber, followed by carbonization, filled the fiber gaps under vacuum. The characteristic peaks for cellulose^[^ ^100^ ^]^ in cattail fiber at ≈3341, 2923, 1737 cm^−1^ disappeared after carbonization, as seen in the FTIR analysis, confirming the effect of carbonization on cattail fiber (Figure 11b). Similarly, the XRD peaks of cellulose in cattail fiber were no longer visible after carbonization, while two new peaks appeared at 22.6 and 43.8° (Figure 11c).^[^ ^99^ ^]^ These new peaks correspond to graphene structures.^[^ ^101^ ^]^ The additional XRD peak at (2θ ∼) 11.8° in PDMS encapsulated cattail fiber confirms the incorporation of PDMS onto cattail fibers (Figure 11c). The cattail fiber‐based pressure sensor responded to pressure loads ranging from 50 to 500 g and to bending angles from 5 to 15° when characterized using warning light, with no noticeable change observed even after 380 cycles, suggesting the outstanding durability of cattail fibers for sensor applications.^[^ ^99^ ^]^

Cattail has also been investigated for fabricating gas sensors to detect phenolic compounds by converting waste cattail fibers into carbon fibers (CFs) using carbonizing process incurred at 500 °C at a heating rate of 5 °C min^−1^ as shown in Figure 11d.^[^ ^102^ ^]^ The gas sensor manufactured from cattail fibers were characterized using deionized water and an interdigital electrode, followed by the formation of the sensing film on the interdigital electrode and drying at room temperature (24 h) (Figure 11e). The cattail fiber‐based gas sensor exhibited exceptional selectivity in detecting phenolic vapor, with its response exceeding ten times than several common atmospheres (Figure 11f), suggesting its viability for selective gas sensing applications. The high sensitivity and selectivity of the cattail fiber‐based gas sensor is believed to be due to the formation of a highly defective graphene structure during carbonization, which causes an increase in reactive sites via pore formation and element doping. In addition, carbon fibers (CFs) obtained from cattail facilitates carrier transport and enable strong responses to aromatic vapors due to π–π interactions with benzene rings, leading to enhanced selectivity to phenolic vapor (Figure 11g).^[^ ^102^ ^]^

### 6.3 Solar Evaporator and Energy Storage

**Figure 12:**
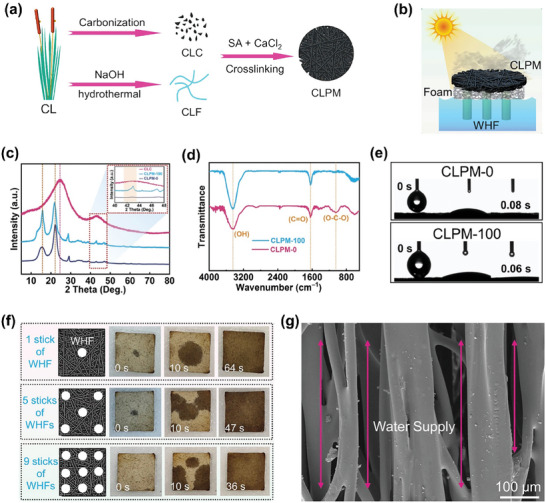
Solar evaporator by Cattails. a) Fabrication of cattail leaf‐based photothermal membrane b) constituent of CLPM‐based solar evaporator c) XRD spectra of CLC, CLPM, and CLPM d) FTIR spectra of CLPM e) hydrophilicity of CLPM f) diffusion capacity of water on the CLMP using different quantities of waste humidifier filter g) morphology structure of waste humidifier filter (a–g: reproduced with permission.^[^ ^105^ ^]^© 2023 Elsevier Ltd.).

International cooperation is crucial to address energy shortages, environmental contamination, and freshwater scarcity. Desalinating seawater, comprising 97% of earth's water, can mitigate global water scarcity.^[^ ^103^ ^]^ However, large amounts of energy is required for conventional desalination techniques, such as thermal distillation and reverse osmosis. Interfacial solar‐driven seawater desalination (ISSD) is a result of the growing demand for water treatment technologies due to its reliability, low cost, high efficiency, and abundant clean energy base.^[^ ^104^, ^105^ ^]^ Cattail leaves and fibers have been investigated for developing solar evaporator device, which consists of a cattail leaf‐based photothermal membrane (CLPM), waste humidifier filter (WHF), and discarded packaging foam.^[^ ^105^ ^]^The CLPM in this study was fabricated by crosslinking cattail leaf‐based fibers CLF) and cattail leaf‐based carbon (CLC) using NaC_6_H_7_O_6_ and CaCl_2_ (**Figure** **12**a,b).

The fabricated CLPM exhibited XRD peaks for cellulose at ≈16 and 22°, while no visible peak is observed for CLC before cross‐linking at ≈16° (Figure 12c). Also, the CLC exhibited broad XRD peaks at ≈23 and 43°, indicating the presence of amorphous carbon.^[^ ^106^ ^]^The hydrophilicity of CLPM was confirmed by the peaks observed at 3380, 1620, and 1040 cm^−1^ during FTIR analysis (Figure 12d) which is in agreement with the results obtained from contact angle measurements (Figure 12e). As shown in Figure 12f, an increase in WHFs results in an increase in water diffusion in the CLPM, preventing salt deposition and ensuring stable desalination performance in ISSD. This could be due to the vertically arranged fibers in the WHF that create channels and facilitate water transport to the surface through capillary action (Figure 12g). Overall, the synergistic effect of amorphous carbon and cellulose facilitates solar steam generation by absorbing sunlight and aiding water transport, respectively, suggesting the potential of cattail leaves as solar evaporators to address freshwater scarcity and energy shortages and to reduce pollution.

Furthermore, cattail fibers (CFs) were also investigated for developing a 3D porous solar evaporator fabricated by applying a polypyrrole (PPy) coating to the CF foam surface.^[^ ^104^ ^]^ The distinct grooved structure of cattail fiber allows the CF foam to have an exceptional capacity for transporting water. Following the application of the PPy coating, the as‐prepared PPy@CF foam exhibits excellent light absorption, good salt resistance, and low heat conductivity. The PPy@CF foam demonstrated exceptional desalination and wastewater purification capabilities, meeting the WHO (World Health Organization) standard for drinkable water.

**Figure 13:**
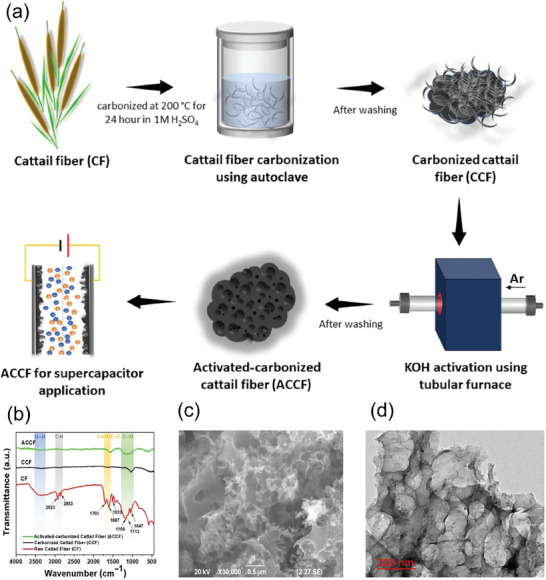
Cattail fibers in energy storage applications. a) The schematic illustration of carbonization and activation process of cattail fiber b) FT‐IR spectra of CF, CCF, and ACCF c) SEM image of the ACCF sample d) TEM image of ACCF material (Reproduced with permission,^[^ ^32^ ^]^ copyright 2024 John Wiley & Sons Ltd.).

Cattails were investigated for energy storage applications by synthesizing activated carbon (AC) from narrowleaf cattail (Typha Angustifolia) fiber through hydrothermal carbonization using sulphuric acid, followed by chemical activation using KOH.^[^ ^32^ ^]^ Cattail fiber worked as a carbon precursor for the synthesis of porous AC. The manufacturing of AC using cattail fiber is shown schematically in **Figure** **13**a. Before carbonization, the fibers were extracted from the leaves and cleaned using 1 M of HCl, deionized water and CH_3_CH_2_OH, followed by drying at 60 °C for 24 h. FTIR spectra of cattail fiber (CF), carbonized cattail fiber (CCF), and activated‐carbonized cattail fiber (ACCF) is shown in Figure 13b. Successful carbonization in activated carbonized cattail fiber (ACCF) was confirmed by the removal of functional groups, such as hydroxyl (‐ OH) groups and CH stretching vibration of cellulosic cattail fiber, after carbonization. The ACCF exhibited uneven, rough (Figure 13c), and porous (Figure 13d) surface structure, which is ascribed to porosity caused by KOH during chemical activation. This study demonstrated the potential of cattail fibers for efficient and cost‐effective energy storage devices in clean energy vehicles, owing to their porous structure and chemical stability. This also positions cattail biomass as a promising source to scale up the carbon materials manufacturing, enabling the conversion of biowaste into cost‐effective energy‐harvesting materials and contributing to the reduction of biowaste accumulation.^[^ ^32^ ^]^

### 6.4 Textile Applications

Global textile production and consumption have increased as a result of population growth and growing living standards.^[^ ^107^ ^]^ According to the Textile‐Exchange report,^[^ ^108^ ^]^ the production of natural plant fibers accounted for ≈30% of the global fiber market, while synthetic fibers made up ≈62%, animal fibers ≈2%, and regenerated fibers ≈6%. Synthetic fibers are stronger, resistant to corrosion, lightweight (ρ_polyester_: 1.4 g/cc versus ρ_cotton_: 1.51 g/cc), durable, and cost‐effective.^[^ ^109^ ^]^ Despite all these facts, the rise in the production of synthetic fibers for the textile industries has caused a substantial environmental pollution challenge.^[^ ^110^ ^]^ On the contrary, cotton is better for the environment than synthetic ones because they are biodegradable, retain more moisture (cotton: 8–11%, polyester: 0.4%), are less harmful to human health (less irritation to the skin, and respiratory system), and are made from renewable resources.^[^ ^111^ ^]^ However, the crop production of cotton involves heavy use of pesticides with approximately requiring 2700 L of water for producing one cotton t‐shirt.^[^ ^112^ ^]^

The environmental pollution caused by synthetic fibers and excessive water usage required for cotton production and processing has led researchers to explore abundant and indigenous natural resources for textile applications.^[^ ^113^, ^114^ ^]^The wetland cattail species *Typha australis* was preliminarily investigated using a chemical retting process to understand its feasibility for textile applications,^[^ ^59^ ^]^ which concluded that cattail fibers have potential to replace traditional natural textile fibers. Further research suggested that cattail fibers exhibited comparable dye exhaustion (≈80%), color‐fastness to heat, physical, and mechanical properties when compared with commonly used textile fibers, such as cotton, flax, hemp, wool, and polyester.^[^ ^11^ ^]^ However, cattail fibers are too rigid to be spun into yarn. The higher stiffness of the cattail fibers is believed to be due to the presence of calcium oxalate plates (COPs) and thick lignified secondary cell walls. Following this the hand of the *Typha* fiber by modifying the surface aerenchyma was investigated in a study^[^ ^22^ ^]^ where COPs, and thick lignified secondary cell walls, concluded that the application of humectant (glycerol) on virgin and bleached fibers improved fiber hand, and significantly increased moisture regain. However, mechanical properties of the fibers decreased substantially. Glycerol‐treated bleached cattail fibers have shown promise to be used in wet‐laid nonwoven applications, such as hygiene tissue, baby wipes, wound dressings, due to their increased moisture absorption properties. In addition, the methods to make spinnable yarn from cattail fibers by blending cattail with cotton at different ratios (cattail: cotton = 0:100, 20:80, 35:65, 50:50) was examined in previous studies.^[^ ^78^ ^]^ This study shows that cotton and cattail fibers can be spun together, with enhancement in consistency when compared to pure cotton yarn. Maximum strength (779.4 CN) was achieved when the cattail to cotton blending ratio was 20:80. Further research is required to unlock the potential cattail fibers for versatile textile applications.

### 6.5 Healthcare Applications

Natural fibers, such as cotton, flax, silk, and linen, are typically used in healthcare textiles, including but not limited to protective textiles, hygienic products, external devices, implantable materials, and extracorporeal devices.^[^ ^115^, ^116^, ^117^ ^]^ However, the applications of these fibers are confined due to their limited antimicrobial properties. Cattail fibers, being a newer renewable resource, have shown versatility for healthcare applications. Antimicrobial composites in medical applications from cattail fibers were developed when treated with benzalkonium chloride and silver nanoparticles.^[^ ^116^ ^]^ Treated cattail fibers demonstrated antimicrobial efficacy against Staphylococcus aureus and Escherichia coli bacteria, as evidenced by the presence of inhibitory halos of ≈11.3 mm. Furthermore, cattails have been investigated for manufacturing composite webs for surgical gowns when blended with polylactic acid (PLA).^[^ ^118^ ^]^ The results of the characterization revealed that cattails exhibited partial antibacterial activity against S. aureus (≈7.78% bacterial reduction), while being neutral against K. pneumoniae. A ratio of 30:70 was recommended for fiber‐to‐PLA content, with suggestions for future improvements in web thickness and resistance properties.

A wound‐healing bandage was developed using *Typha elephantina*, which exhibited anti‐inflammatory and wound‐healing activities due to its β‐sitosterol and quercetin content.^[^ ^119^ ^]^ The high tensile strength of wound healing bandages incorporated with *Typha* was confirmed while being experimented on in a wistar rat, which is believed to be due to the promotion of collagen. *Typha* methanolic extracts, made using the disc diffusion method, exhibited strong and moderate antibacterial activity against E. coli and Staphylococcus aureus, respectively.^[^ ^120^ ^]^ Apart from medical textiles, *Typha* has been shown to have several medication uses. For instance, *Typha angustata* for nasal bleeding, hemotemsis, hemomaturia, uterine bleeding, dysmenorrhea, postpartum abdominal pain, gastralgia, scrofula, and abscesses^[^ ^52^ ^]^ and enhancement of demineralized bone matrix^[^ ^121^ ^]^ and *Typha domingensis* for treating gastrointestinal diseases and metabolic disorders.^[^ ^122^, ^123^ ^]^

Cattails have sedative and tranquilizing properties that help both adults and children by reducing body temperature and lowering blood pressure.^[^ ^37^ ^]^ Cattails are also useful to promote uterine contractions, wash ulcers, and remove tumors from the mouths of children with thrush.^[^ ^42^ ^]^ Apart from the leaves, cattail flowers are valued for their therapeutic properties in treating burns. The male inflorescence is specifically used as a topical application for wounds and ulcers.^[^ ^42^ ^]^ The most recent development of cattails in healthcare textiles is the production of sanitary menstrual pads, aiming to reduce plastic waste while ensuring comfort and functionality.^[^ ^124^ ^]^This project demonstrated the sustainability and effectiveness of cattail fibers in medical and hygienic products.

### 6.6 Insulation

Heating and cooling facilities in buildings and industries consume ≈40% of global energy, with fossil fuels accounting for ≈70% of this total.^[^ ^125^ ^]^ Coal, diesel generators, and so forth are typically used to meet this energy demand. These fuels pollute the environment, leading to deforestation and greenhouse gas emissions.^[^ ^126^ ^]^ Low‐cost insulating materials can reduce emissions by reducing the heat absorption during summer and heat losses during wintertime.^[^ ^127^, ^128^ ^]^ Petrochemicals, such as polystyrene and some natural resources like glass, rock wools, etc. are conventionally used insulating materials that are energy intensive and detrimental to human health.^[^ ^129^ ^]^ Fiberglass, one of the most commercially‐used insulating materials, used until 1996, caused emphysema and lung cancer.^[^ ^130^ ^]^

Cattail, being an abundant low‐cost natural waste plant fiber, has the potential to be used as an insulator due to its promising insulation characteristics,^[^ ^130^, ^131^, ^132^ ^]^ which is believed to be due to its unique structural configuration filled with soft open‐cell tissue structure. Cattail has been investigated for manufacturing insulation boards from narrow‐leaved cattail fibers using hot pressing and Methylene Diphenyl Diisocyanate (MDI) as a binder.^[^ ^130^ ^]^ Numerous studies on naturally occurring insulating materials, such as maize husk, groundnut shell, coconut pith and paddy straw, cotton seed hulls, durian peel and coconut coir, eggplant stalks, tamarind hulls, and cotton stalk fibers, have been conducted to identify alternatives to petrochemicals as a source for building insulation.^[^ ^130^, ^133^ ^]^ The thermal conductivity of the insulation boards manufactured from cattail fibers varied between 0.0438 and 0.0606 Wm^−1^ K^−1^, which is typically less than that of the commonly used biobased materials in insulation, such as wheat straw (0.0481–0.0521 Wm^−1^ K^−1^) and cotton stalk fibers (0.0585–0.0815 Wm^−1^ K^−1^). This suggests that the cattail boards are more efficient in preventing heat transfer than conventional biobased insulation materials. In another study, the magnesite‐bound *Typha* board exhibited a thermal conductivity of ≈0.055 Wm^−1^ K^−1^, a thermal transmittance coefficient of ≈0.35 Wm^−^^2^ K^−1^, with a very high resistance to mold growth.^[^ ^134^ ^]^ The thermal conductivity of different cattail‐based insulating materials is summarized in **Table** **7**.

**Table 7:** Thermal conductivity of different cattail‐based insulating materials.

Cattail‐based insulating Material	Thermal conductivity [W/mK]	References
Magnesite‐bound *Typha* board	0.055 W/mK	[132]
Insulation Cattail fiberboard with Methylene Diphenyl Diisocyanate binder, density 200–400 kg m^3^	0.0438–0.0606 W/mK	[130]
The magnesite‐bound *Typha* board.	0.052 W/mK	[135]
Mixture of Cattail powder leaves and Gum arabic binder	0.055 to 0.083 W/mK	[131]

### 6.7 Bioenergy Resource

Renewable transportation fuels are being developed to increase energy security and reduce greenhouse gas (GHG) emissions. Renewable energy derived from natural biomass could substitute petroleum goods while mitigating GHG emissions and increasing the value of the land. Although ethanol derived from starch and biodiesel is still the most widely used, cellulose conversion technology from natural biomass is advancing.^[^ ^118^ ^]^ Cattail biomass has the potential to be utilized as a bioenergy resource. Producing bioethanol from the cattails is one way to increase land value while minimizing environmental impact. Ethanol produced from cattail biomass offers a ≈90% reduction in carbon emissions when compared to gasoline, while starch‐based ethanol offers ≈29% reduction.^[^ ^136^, ^137^ ^]^ Cattail yields from constructed wetlands vary between ≈16.1 and 42.7 metric ton/ha, with a ≈43.4% bioethanol conversion efficiency. However, the addition of conversion organisms could enhance conversion efficiency. Harvesting Typha together with glauca in great lakes wetlands could potentially substitute detrimental methods like herbicide use and burning, aiding ecological restoration and bioenergy production.^[^ ^25^ ^]^ Cattail biomass also shows promise for biogas production, with methane yields of 60% from fresh and 57% from dry biomass, highlighting its potential for energy and chemical synthesis.^[^ ^138^ ^]^

## 7 Future Directions and Outlook

**Figure 14:**
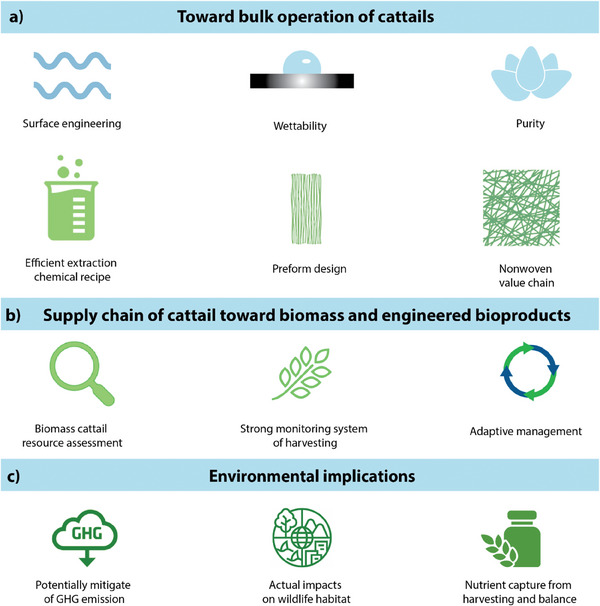
Future directions for cattail biomass. a) Toward bulk operation of cattails. b) Supply chain of cattail toward biomass and engineered bioproducts. c) Environmental implications.

The future directions and outlook of cattail biomass are shown schematically in **Figure** **14**. The requirements of sustainable materials (reusable, recyclable, and biodegradable) as a replacement for hazardous synthetic materials are catching the attention of scientists and researchers worldwide. The leaves and extracted fibers from the different parts of the abundant wetland plant cattail show promising characteristics and properties for use in a wide range of applications. Researchers have extracted the fibers by water retting, mechanical, or chemical means according to the end use, as the physical and mechanical properties depend on the extraction method of the fibers. For water retting, the fiber yield has been found to be 8.95%; whereas chemical extraction by alkali treatment has resulted in a fiber yield of 46.6%. The hollow structure, porosity, and lighter‐weight nature make cattail suitable for use as biocomposites, sensors, solar evaporators, textiles, healthcare, insulation, and bioenergy resources.

However, future studies should focus on surface engineering of cattail fibers to reduce fiber density and maximize wettability, fiber's hand (surface smoothness), flexibility, and purity. The former two, i.e., reducing fiber density and maximizing wettability, are required to manufacture lighter‐weight and high‐performance composites, while the latter three, i.e., maximizing fiber's hand, flexibility, and purity, are required to convert cattail fibers into spinnable yarns (Figure 14a). Future research must focus upon developing an exact chemical formulation and an appropriate chemical treatment procedure for the efficient extraction of calcium oxalate plates (COPs) from cattail fibres.

Currently, there are no nonwoven suppliers in Canada. The abundance of the cattails should be utilized by startup companies and industries to create a value chain for large scale fiber production and nonwoven manufacturing. Further work is needed to control the web thickness during mat manufacturing. In the future, more thorough research will be needed to create biodegradable composites with cattail and biodegradable matrices. The growth of cattails should be controlled effectively without causing any detrimental effects on the environment. This can result in an efficient CO_2_ sequestration while simultaneously creating new market opportunities by collaborating efficiently with local and state governments and industry alliances. An adaptive management framework should be developed using current and upcoming studies as an instructional resource for harvesters, processors, and the government. The implementation of cattail harvesting will be closely observed, and the positive and negative results will be quantified, owing to a robust monitoring system (Figure 14b). This will allow for long‐term environmental implications (Figure 14c) such as the amount of greenhouse gas emissions that may be reduced by harvesting cattails and other ecological biomass. The actual effects on wildlife habitat and the strategies for reducing any adverse effects can be measured. The long‐term retention of nutrients after harvesting and the balance of nutrients in harvested areas will be possible to control.^[^ ^139^ ^]^

However, there is significant potential for future investigations into cattail fibers, especially for bioengineered products, presenting both challenges and opportunities. The inconsistent fiber quality resulting from different growing environments and extraction methods is a major problem. To decide whether it is suitable for industrial application, more precise and uniform mechanical data is required, including durability and tensile strength. Scaling up fiber extraction processes and integrating *Typha* fibers into existing manufacturing systems remain technical hurdles. Additionally, environmental and economic feasibility, including land use and water consumption, must be evaluated to ensure sustainability. However, *Typha* fibers offer great opportunities in several sectors. Their lightweight, biodegradable nature makes them promising candidates for bio‐composites in automotive, construction, and packaging industries, as well as for sustainable textiles and nonwoven materials. They could also be utilized in green building materials, biodegradable plastics, and functional bio‐products like water filtration and insulation materials. By addressing these challenges through standardization, scalable processing, and environmental assessments, future research could unlock the potential of Typha fibers, promoting the development of sustainable, bioengineered products that contribute to a circular economy and reduce environmental impact.

## 8 Conclusion

This comprehensive review demonstrates the potential of cattail fibers across various fields. By summarizing its species, fiber extraction techniques, and process optimizations, alongside the characterization and properties of *Typha* fibers, we provided a thorough understanding of its capabilities. The environmental benefits of *Typha*, particularly in reducing carbon footprint and greenhouse gas emissions, underscore its viability as a sustainable alternative material. This study highlights how *Typha* fiber, when managed thoughtfully, can contribute to resource‐efficient technologies, transforming waste into value‐added products and fostering a circular economy. With further research and development, *Typha* fibers can play a significant role in sustainable practices, offering a renewable, resilient, and abundant biomass source. This not only promotes waste reduction and responsible consumption but also encourages innovation in renewable material development. The insights from this review can expedite progress toward a more sustainable future, where the utilization of natural resources is both efficient and environmentally friendly.

## Conflict of Interest

The authors declare no conflict of interest.
